# Updated HIV-1 Consensus Sequences Change but Stay Within Similar Distance From Worldwide Samples

**DOI:** 10.3389/fmicb.2021.828765

**Published:** 2022-01-31

**Authors:** Gregorio V. Linchangco, Brian Foley, Thomas Leitner

**Affiliations:** Theoretical Biology and Biophysics Group, Los Alamos National Laboratory, Los Alamos, NM, United States

**Keywords:** HIV, subtypes, consensus sequences, evolution, molecular epidemiology, pandemic

## Abstract

HIV consensus sequences are used in various bioinformatic, evolutionary, and vaccine related research. Since the previous HIV-1 subtype and CRF consensus sequences were constructed in 2002, the number of publicly available HIV-1 sequences have grown exponentially, especially from non-EU and US countries. Here, we reconstruct 90 new HIV-1 subtype and CRF consensus sequences from 3,470 high-quality, representative, full genome sequences in the LANL HIV database. While subtypes and CRFs are unevenly spread across the world, in total 89 countries were represented. For consensus sequences that were based on at least 20 genomes, we found that on average 2.3% (range 0.8–10%) of the consensus genome site states changed from 2002 to 2021, of which about half were nucleotide state differences and the rest insertions and deletions. Interestingly, the 2021 consensus sequences were shorter than in 2002, and compared to 4,674 HIV-1 worldwide genome sequences, the 2021 consensuses were somewhat closer to the worldwide genome sequences, i.e., showing on average fewer nucleotide state differences. Some subtypes/CRFs have had limited geographical spread, and thus sampling of subtypes/CRFs is uneven, at least in part, due to the epidemiological dynamics. Thus, taken as a whole, the 2021 consensus sequences likely are good representations of the typical subtype/CRF genome nucleotide states. The new consensus sequences are available at the LANL HIV database.

## Introduction

In 2020, 37.7 million people worldwide were living with HIV, of which 1.5 million became infected in 2020. Until 2020, 36.3 million people have died from AIDS-related illnesses (UNAIDS, 2021). Most of these infections are by HIV-1. The burden of HIV is uneven across the world, between countries, within and between risk groups, and between ethnic groups in different geographical regions. In large due to founder effects, different genetic variants, i.e., subtypes and circulating recombinant forms (CRFs), have spread unevenly across the world (Hemelaar et al., 2019, 2020).

While analyses of individual HIV sequences provides comprehensive information about worldwide and local epidemics as well as detailed information about within-host evolution, global reference sequences have many uses. One type of reference sequences is consensus sequences, i.e., a sequence that represents the most commonly found nucleotide (or amino acid) at each site. Such sequences are useful as references for bioinformatic processing in, for instance, alignments and contig assembly, for detection of hypermutants, gene detection and annotation, and for representing simplified views and data from complex populations (Rose and Korber, 2000; Lee, 2003; Seah et al., 2020; Domingo et al., 2021; Frith et al., 2021; Kulikova et al., 2021; Zhang et al., 2021). Consensus sequences have also been used in studies of protein functions, binding, and vaccine designs (Novitsky et al., 2002; Gao et al., 2005; Nickle et al., 2007; Yan et al., 2007; Sternke et al., 2019).

**FIGURE 1:**
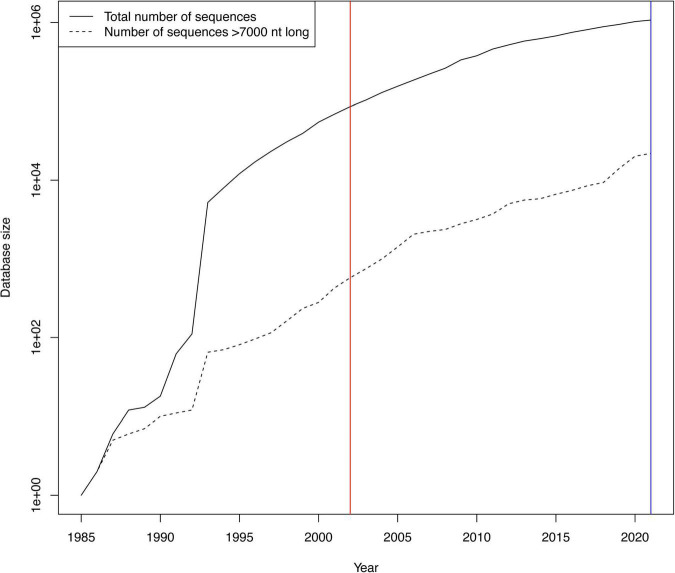
Growth of HIV-1 sequences in the LANL HIV database. The growth of the number of publicly available HIV sequences has been roughly exponential since the beginning of the HIV era. The y-axis is logarithmic to make the near full genome (>7,000 nt) sequence count visible. The red vertical line shows when the last previous HIV consensus sequences were calculated in 2002, and the blue line when we calculated the new ones in this publication in 2021.

The LANL HIV database (Foley et al., 2018) provides global consensus sequences for HIV-1 subtypes and CRFs. The most recent genome level consensus sequences are from 2002 (and some gene specific consensus sequences from 2004). Since 2002, the number of available sequences in the database has grown exponentially, from 85,926 to 1,073,050 in 2021, a >12-fold increase (Figure 1). Similarly, near full length genomes (sequences > 7,000 nt long) have increased from 574 to 21,952, a massive > 38-fold increase. Over this time, sequencing of non-EU and non-US samples has increased the most, and thus the increase mostly reflects HIV-1 sequences from the rest of the world, where most of the infected people live (UNAIDS, 2021). Therefore, it is necessary to re-evaluate the global consensus sequences.

## Materials and Methods

### Sequence Data

To generate new HIV-1 consensus sequences, we used the LANL HIV database 2019 filtered web alignments of full genomes, available at https://www.hiv.lanl.gov/content/sequence/NEWALIGN/align.html. This alignment is a high-quality selection of the complete 2019 web alignment. The sequences in this set have no or only one minor frameshift, <1% nucleotide ambiguities, no nucleotide ambiguities that affect translation, and no unusual indels. This set was considered ideal for global consensus sequence generation. This set contained 4,312 sequences. For comparison to our new consensus sequences, we used the latest previously calculated consensus sequences, from 2002, also available at https://www.hiv.lanl.gov/content/sequence/NEWALIGN/align.html.

To evaluate how distant actual HIV-1 genomes are from the consensus sequences, we included (1) HIV-1 genome sequences with >7,000 nt, (2) sequences that have a sampling year, (3) sequences that were not labeled as “problematic” in the LANL HIV database (see https://www.hiv.lanl.gov/components/sequence/HIV/search/help.html for an explanation of what “problematic” means), and (4) restricted the data to only include one sequence per patient when >1 sequence was known to come from a patient. This set contained 4,674 sequences, accessed 2021-06-23.

### Consensus Calculation

Consensus sequence calculations were performed with the Advanced Consensus Maker, available at https://www.hiv.lanl.gov/content/sequence/CONSENSUS/AdvCon.html. We used a minimum of three sequences per HIV-1 subtype or circulating recombinant form (CRF) to generate new consensus sequences (reducing the number of useable sequences to 3,470 from the 2019 web alignment of 4,312 sequences), a majority rule that assigns the most common nucleotide state to each site, tie-breaking that follows the typical nucleotide frequency in HIV-1 sequences (i.e., priority in order A, G, T, C), and no gap removal. These settings are the current defaults for these consensus calculations, and have been used for the previous consensus sequence calculations at the LANL HIV database.

### Sequence Comparisons

**FIGURE 2:**
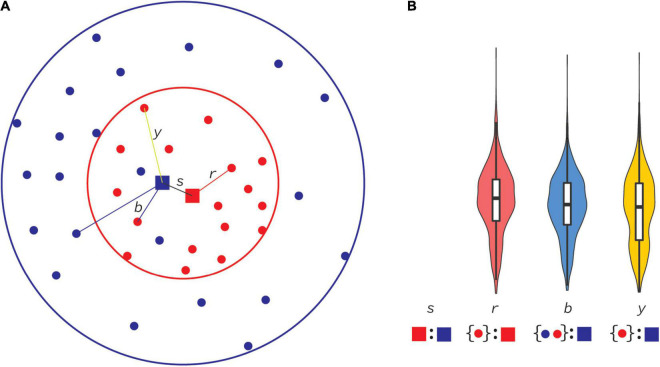
Principal distances between consensus and database sequences. In this cartoon, sequences evolve through mutations over time, radiating out from the origin (center of circles) and sampled through time **(A)**. At some time (red circle) all sequences sampled until then (red dots) are used to compute a consensus sequence (red square). Individual distances from that consensus sequence to all sequences available until then form a distance distribution in panel **(B)**, displayed as a red violin plot of all distances *r*. An example of a *r* distance is shown in panel **(A)**. At a later time (blue circle), a new consensus sequence is computed (blue square), and, similarly, all distances (*b*) to sequences available until that time (red and blue dots) form the blue distribution in panel **(B)**. The distance between the first and second consensus sequences is *s*. We can also consider distances from the second consensus sequence to samples collected only available until the first consensus was made (*y*). Note that some samples that originated from a time before the first consensus was made were not publicly available until the second consensus was made (blue dots inside red circle).

Pairwise alignments were made with MAFFT V7 (Katoh and Standley, 2013), followed by codon correction using GeneCutter,^1^ in all sequence comparisons. Pairwise comparisons were performed between previous and new consensus sequences as well as between individual HIV-1 genome sequences (>7,000 nt) and consensus sequences (Figure 2). Each pairwise alignment was then analyzed with a custom python script that counted state changes, insertions, deletions, and sequence length. Flanking gaps in each pairwise alignment were ignored. The R programming environment and ggplot (R Development Core Team, 2003; Wickham, 2016) were used to generate violin plots to display distributions of these categories, and Wilcoxon rank sum tests with Bonferroni multiple-test correction to assess potential differences.

## Results

### Changes in HIV-1 Consensus Sequences

The number of HIV-1 sequences in the LANL HIV database has grown over time (Figure 1). Both the total number of sequences and the number of near full genomes (>7,000 nt) has grown roughly exponentially. The substantial growth of the database since 2002, when genome level consensus sequences were last updated, motivated us to assess potential changes in the consensus sequences. In total, 90 new HIV-1 subtype or CRF consensus sequences were generated based on at least three available near full genome sequences in each such set (Supplemental Results). Out of those, 18 subtypes/CRFs (and CPZ) allowed for comparison between the 2002 and 2021 consensus sequences (Table 1). In 2002, only four of these subtype consensus sequences were based on a substantial number of sequences (A1, B, C, and D used > 30 sequences), while the rest used <10 sequences each. In 2021, nearly all used substantial numbers; subtypes B and C, the two most sequenced subtypes in the database, used 1,294 and 744 sequences, respectively, for the 2021 consensus sequences. Typically, the 2021 consensus sequences were shorter than in 2002, i.e., they had more “deletions” than “insertions” relative to the 2002 consensuses. Typically, there were also many “substitutions” between the 2002 and 2021 consensuses, on average 109 nucleotide state differences across the entire genome (1.1%), excluding HIV-1 group O and CPZ consensuses, which had more. Overall, counting all indel and nucleotide state differences (including those in group O and CPZ), on average 2.3% (range 0.8–10%) of the consensus genomes changed from 2002 to 2021.

**TABLE 1:** 2002–2021 HIV-1 consensus sequence comparison.

Subtype/CRF	Insertions	Deletions	Substitutions	N seq used in cons 2002	N seq used in cons 2021	N genome seq in 2002	N genome seq in 2021
A1	3	10	60	40	173	57	188
B	3	403	96	31	1,294	326	2,024
C	6	35	56	66	744	189	1,214
D	0	25	68	33	71	53	77
F1	17	23	135	4	42	12	73
G	9	22	205	5	80	21	85
H	16	4	221	3	10	8	10
O	24	97	401	4	49	35	45
01_AE	4	110	52	9	350	122	636
02_AG	4	66	94	7	130	49	160
04_CPX	29	13	109	3	5	5	5
06_CPX	10	21	118	4	11	4	11
07_BC	1	46	86	3	22	2	38
08_BC	6	12	121	4	21	8	33
10_CD	15	16	51	3	3	3	3
11_CPX	8	20	149	6	22	12	23
12_BF	20	10	53	6	9	12	15
14_BG	27	7	91	6	5	8	12
CPZ	181	62	736	5	21	7	18

*Insertions, deletions, and substitutions are relative differences comparing 2002–2021 consensus sequences.*

**FIGURE 3:**
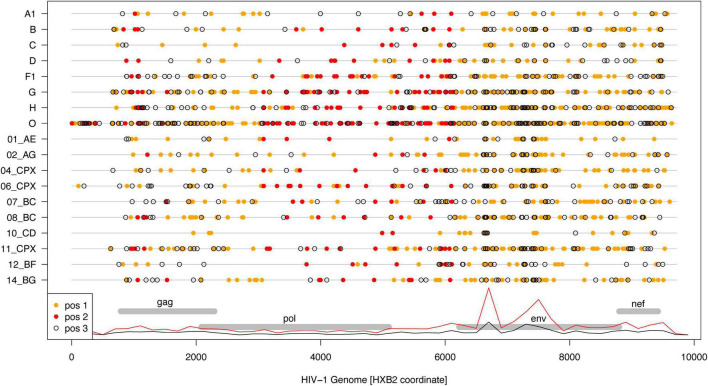
Differences between 2002 and 2021 HIV-1 subtype/CRF consensus sequences. Dots show nucleotide state differences for 1st (orange), 2nd (red), and 3rd (open) codon positions along the respective subtype/CRF genome (gray lines). The genome locations for the structural genes are shown for reference, and overlayed with relative densities of 1st + 2nd (red) and 3rd (black) codon position differences across all subtypes/CRFs.

Interestingly, non-synonymous “substitutions” dominated in the 2002 to 2021 consensus comparisons (Figure 3). Overall, “substitutions” in codon positions 1 and 2 were about 3.5 times more frequent than in codon position 3. This result should not be surprising because the “substitutions” (as well as “insertions” and “deletions”) are simply differences between the 2002 and 2021 consensus sequences, which are manmade constructs not only reflecting evolutionary processes but also sampling effects. On the other hand, most nucleotide state differences (“substitutions”) occurred in *env*, and least in *pol* (Figure 3), which is expected from the known differences in the evolutionary rate across the HIV-1 genome.

### Consensus Sequences Remain Equally Distant From Worldwide Sequences Over Time

**FIGURE 4:**
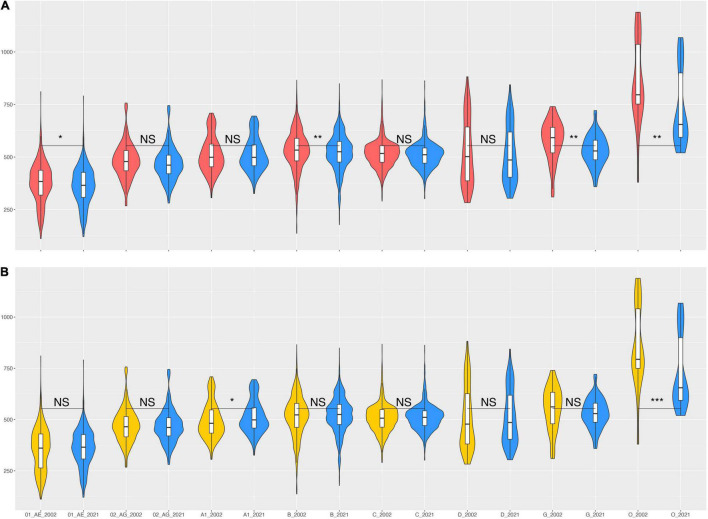
Nucleotide state differences between HIV-1 consensus sequences and individual HIV-1 genomes from across the world. **(A)** Violin plots of the distribution of nucleotide state differences between individual HIV-1 sequences sampled up until 2002 and the 2002 consensuses (red) and individual HIV-1 sequences sampled up until 2021 and the 2021 consensuses (blue). **(B)** Violin plots of the distribution of nucleotide state differences between individual HIV-1 sequences sampled up until 2002 and the 2021 consensuses (yellow) and, again, individual HIV-1 sequences sampled up until 2021 and the 2021 consensuses (blue). Violin plot margins show the distribution of possible values, box margins 25% (*Q1*) and 75% (*Q3*) quantiles (*IQR*), box whiskers indicate *Q*1−1.5 × *IQR* and *Q*3 1.5 × *IQR*, the median is depicted by a horizontal line. Pairwise comparisons of the distributions show significance assessed by a two-sided Wilcoxon rank sum test with Bonferroni multiple-test correction (*p* = α/*m*, with α = 0.05(*), α = 0.01(**), α = 0.001(***), and NS = not significant, for *m* = 16 tests).

Even though the consensus sequences have changed since 2002 until 2021 (Table 1), most subtypes/CRFs have stayed within a similar genetic distance to the consensuses over this time span (Figure 4). We compared eight subtypes/CRFs that had at least 20 worldwide genome sequences sampled in 2002 (and 2021). Overall, 2021 consensuses were somewhat closer to the worldwide genome sequences, i.e., showing on average fewer nucleotide state differences, but only subtypes B, G, and group O sequences displayed significant differences (Figure 4A).

To assess whether the changes in the 2021 consensus sequences induced significant differences over time, we compared the 2021 consensus sequences to genome sequences sampled until 2002 or 2021, i.e., the 2021 set had additional sequences that became available after 2002 (“N Genome Seq” columns in Table 1). Again, on average most subtypes/CRFs showed no significant change in their distance to the worldwide sequences available until 2002 or 2021 (Figure 4B). Only group O sequences showed a significant difference. We note that group O consensus sequences had the biggest change from 2002 to 2021 (401 nucleotide state changes) and a 29% growth in available genome sequences (Table 1).

While comparing 2002–2021 consensuses to each other showed more deletions than insertions (Table 1), comparing consensuses to worldwide genome sequences showed the opposite (Supplementary Figures 1, 2). Thus, Subtypes/CRFs 01_AE, 02_AG, B, C, D, and group O had significant changes in insertions, while only 01_AE, B, and C showed significant changes in deletions.

## Discussion

The LANL HIV database has grown exponentially, adding hundreds of thousands of sequences since the 2002 and thousands of full genome sequences that informed the new HIV-1 subtype/CRF consensus sequences in this study (in 2021). The new consensuses differed overall in about 2.3% of the genome, of which about half were nucleotide state differences. Of that, nearly 3/4 were non-synonymous changes, i.e., changes inducing amino acid differences. Such changes may be important for vaccine design and other scientific purposes where protein sequences are important.

As shown in Figure 4, most real-world HIV-1 genome sequences stayed at about the same distance from the 2021 consensuses as they did in 2002. This is explained by the relatively small overall difference between the 2002 and 2021 consensuses as compared to the distances to the real-world genome sequences, i.e., at about 1.1% consensus-to-consensus distance and about 5% consensus-to-real sequence distance. The principle of this is shown in Figure 2. The differences were, however, uneven across many aspects of the data. On the genome level, *env* had most differences because it (mostly the variable loop regions) evolves faster than other parts of the genome. Moreover, for certain uses, a 1% overall genome difference is meaningless because a specific amino acid at a certain site may make all the difference. On the subtype/CRF level, some subtype/CRF consensus sequences changed more than others, ranging from 0.8 to 10% (Table 1), e.g., while CRF01 only changed nucleotide state at 49 sites when going from building consensus sequences based on nine sequences in 2002 to 350 in 2021, subtype H consensus sequences differed at 222 sites going from 3 to 10 underlying sequences.

Consensus sequences are computational constructs rather than real world biological entities. As such, consensus sequences may not exist in nature, yet it has been shown that they may describe stable and representative protein structures (Sternke et al., 2019) that may be suitable for vaccines (Novitsky et al., 2002; Nickle et al., 2007). Furthermore, consensus sequences are affected by potential sampling biases. In our case, worldwide HIV-1 genome sequences have not been randomly sampled, instead they are simply all sequences ever published in the international literature, for whatever purpose. Nevertheless, the new HIV-1 subtype/CRF consensus sequences in this study were based on up to 1,294 observed genome sequences each, and by now most geographical regions of the world have had subtype/CRF surveys, all which contributed near full genome sequences included in these new consensus sequences. Here, 89 countries were included among these sequences. Some subtypes/CRFs have had limited geographical spread, and thus sampling, which is not the same as unrepresentative sampling, is uneven due to the epidemiological dynamics. Two other potential reasons for change from 2002 until 2021 is more use of antiviral drugs in some parts of the world, and changes in sequencing technologies. Recall, however, that the 2021 consensuses include all high-quality sequences, including those used in 2002. Thus, overall, the 2021 consensus sequences likely are good representations of the typical subtype/CRF genome nucleotide states.

Alternatives to consensus sequences include phylogenetically inferred ancestral sequences (Thornton, 2004), the most frequently observed actual sequence in a population, the most central real sequence in a population, and so-called mosaic sequences (Thurmond et al., 2008). Each one of these alternatives are also computational constructs that depend on assumptions related to sampling and evolutionary processes. They may each have their strengths and limitations in whatever use they are put to.

## Conclusion

In conclusion, with the large increase of available full genome sequences from across the world, the 2021 consensus sequences likely are good representations of the typical subtype/CRF genome nucleotide states. The new consensus sequences are available at the LANL HIV database for public use.

## Data Availability Statement

All new HIV-1 consensus sequences calculated in this study are available at https://www.hiv.lanl.gov/content/sequence/NEWALIGN/align.html under the Alignment type “Consensus/Ancestral” type, Year “2021”.

## Author Contributions

GL and TL conceived and designed the study. GL, BF, and TL analyzed the data and wrote the manuscript. All authors contributed to the article and approved the submitted version.

## Conflict of Interest

The authors declare that the research was conducted in the absence of any commercial or financial relationships that could be construed as a potential conflict of interest.

## Publisher’s Note

All claims expressed in this article are solely those of the authors and do not necessarily represent those of their affiliated organizations, or those of the publisher, the editors and the reviewers. Any product that may be evaluated in this article, or claim that may be made by its manufacturer, is not guaranteed or endorsed by the publisher.

## Footnotes

1
https://www.hiv.lanl.gov/content/sequence/GENE_CUTTER/cutter.html
