# Supplementary figures and images for: Updated HIV-1 Consensus Sequences Change but Stay Within Similar Distance From Worldwide Samples
Source: Front Microbiol. 2022 Jan 31;12:828765. doi: 10.3389/fmicb.2021.828765 (PMC8843389; doi:10.3389/fmicb.2021.828765)

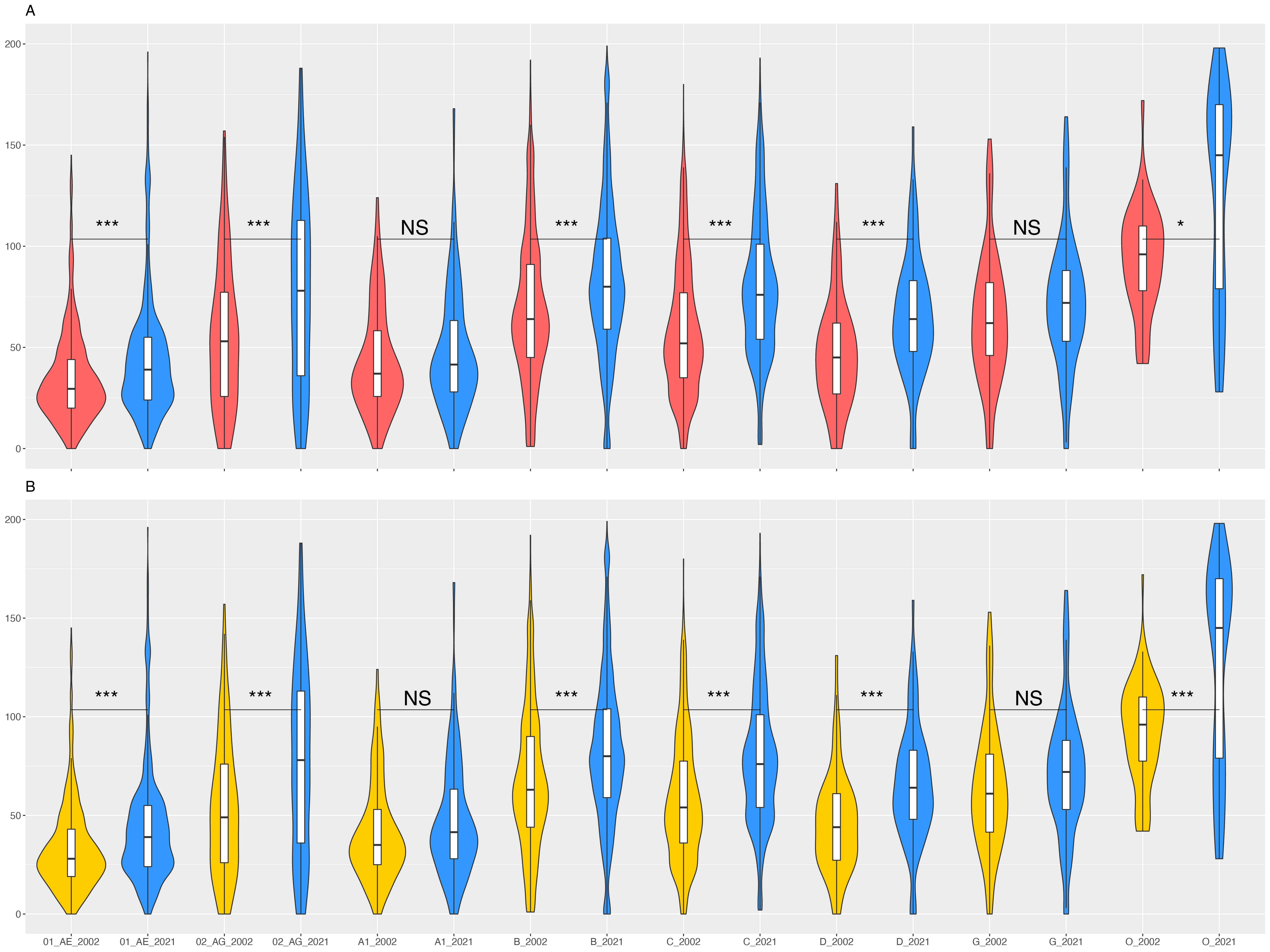

Supplement: Supplementary file 1 [file Image_1.JPEG]

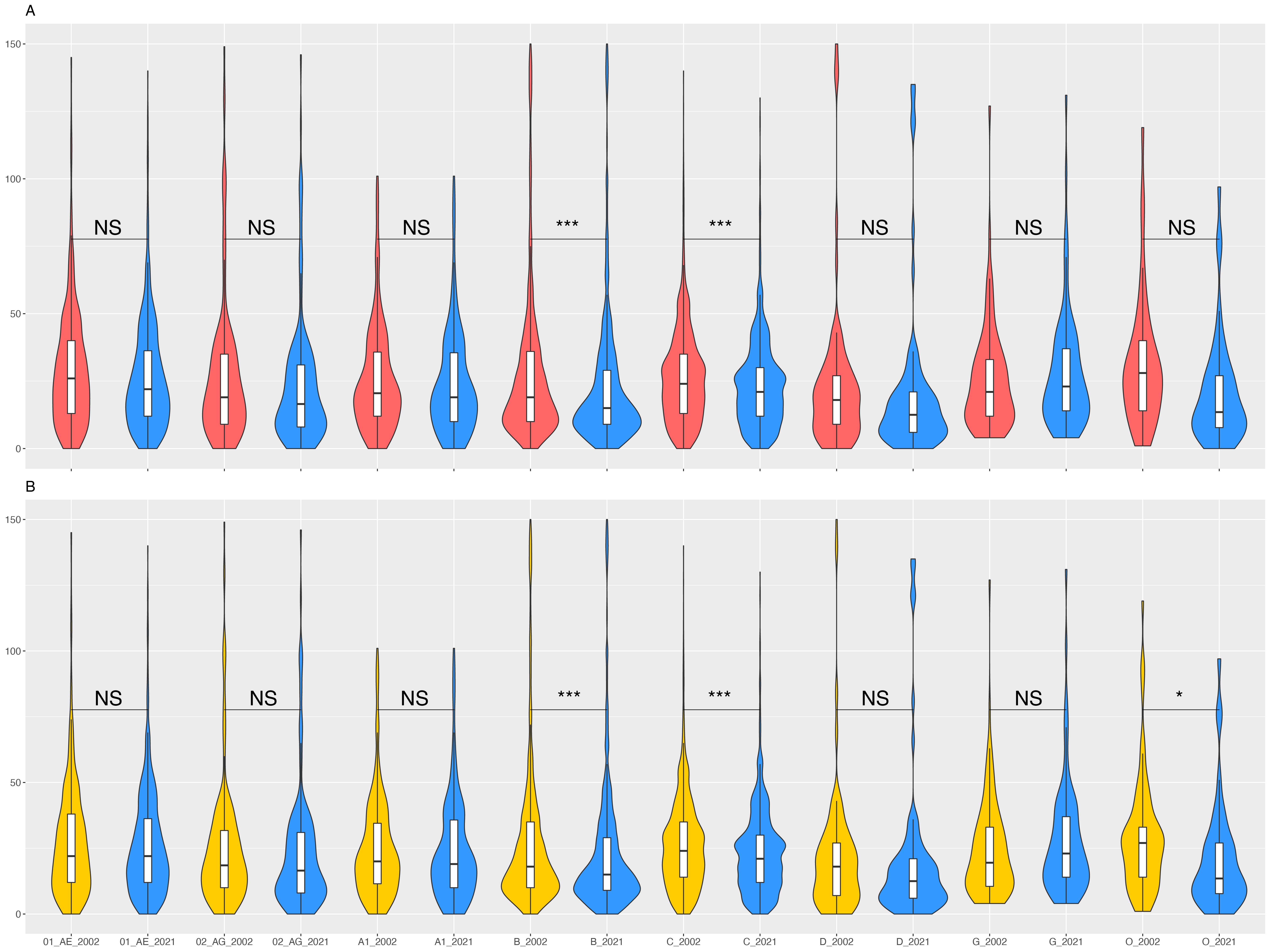

Supplement: Supplementary file 2 [file Image_2.JPEG]
