# Supplementary material for: Updated HIV-1 Consensus Sequences Change but Stay Within Similar Distance From Worldwide Samples
Source: Front Microbiol. 2022 Jan 31;12:828765. doi: 10.3389/fmicb.2021.828765 (PMC8843389; doi:10.3389/fmicb.2021.828765)
Supplement: Supplementary file 3 [file Table_1.DOCX]

**Supplementary materials for:**

**“Updated HIV-1 consensus sequences change but stay within similar distance from worldwide samples”**

Gregorio Linchangco, Brian Foley, Thomas Leitner*

Theoretical Biology and Biophysics group, Los Alamos National Laboratory, Los Alamos, New Mexico, USA

*** Correspondence:**Corresponding Author
tkl@lanl.gov

**TABLES**

**Supplementary Table 1. List of 90 newly constructed consensus sequences for 2021 and the number of sequences used to generate them.**

| **Subtype/CRF** | **N Sequences** | **Subtype/CRF** | **N Sequences** | **Subtype/CRF** | **N Sequences** | **Subtype/CRF** | **N Sequences** |
| --- | --- | --- | --- | --- | --- | --- | --- |
| 01_AE | 350 | 28_BF | 5 | 57_BC | 7 | 99_BF | 3 |
| 02_AG | 130 | 29_BF | 7 | 58_01B | 5 | 100_01C | 3 |
| 04_cpx | 5 | 31_BC | 3 | 59_01B | 4 | 103_01B | 3 |
| 05_DF | 4 | 33_01B | 6 | 60_BC | 4 | A1 | 173 |
| 06_cpx | 11 | 35_AD | 19 | 63_02A | 3 | A2 | 4 |
| 07_BC | 22 | 36_cpx | 3 | 64_BC | 7 | A3 | 3 |
| 08_BC | 21 | 37_cpx | 4 | 65_cpx | 4 | A6 | 67 |
| 09_cpx | 5 | 38_BF1 | 4 | 66_BF1 | 3 | B | 1295 |
| 10_CD | 3 | 39_BF | 3 | 69_01B | 5 | C | 744 |
| 11_cpx | 22 | 40_BF | 4 | 70_BF1 | 3 | D | 71 |
| 12_BF | 9 | 43_02G | 4 | 71_BF1 | 12 | F1 | 42 |
| 13_cpx | 10 | 44_BF | 3 | 72_BF1 | 5 | F2 | 10 |
| 14_BG | 5 | 45_cpx | 5 | 74_01B | 3 | G | 80 |
| 15_01B | 8 | 46_BF | 8 | 78_cpx | 3 | H | 10 |
| 16_A2D | 4 | 47_BF | 3 | 85_BC | 8 | J | 5 |
| 17_BF | 7 | 48_01B | 3 | 86_BC | 3 | K | 3 |
| 18_cpx | 6 | 49_cpx | 4 | 87_cpx | 3 | L | 3 |
| 19_cpx | 5 | 50_A1D | 4 | 88_BC | 3 | N | 11 |
| 21_A2D | 3 | 51_01B | 3 | 90_BF1 | 6 | O | 49 |
| 22_01A1 | 8 | 52_01B | 3 | 92_C2U | 5 | CPZ | 21 |
| 24_BG | 3 | 53_01B | 4 | 93_cpx | 3 | GOR | 6 |
| 25_cpx | 5 | 54_01B | 3 | 95_02B | 3 |  |  |
| 26_A5U | 4 | 55_01B | 4 | 96_cpx | 3 |  |  |

**FIGURE LEGENDS**

**Supplementary Figure 1.** **Insertions between HIV-1 consensus sequences and individual HIV-1 genomes from across the world. (A)** Violin plots of the distribution of nucleotide insertions between individual HIV-1 sequences sampled up until 2002 and the 2002 consensuses (red) and individual HIV-1 sequences sampled up until 2021 and the 2021 consensuses (blue). **(B)** Violin plots of the distribution of nucleotide insertions between individual HIV-1 sequences sampled up until 2002 and the 2021 consensuses (yellow) and, again, individual HIV-1 sequences sampled up until 2021 and the 2021 consensuses (blue). Violin plot margins show the distribution of possible values, box margins 25% ($Q1$) and 75% ($Q3$) quantiles ($IQR$), box whiskers indicate $Q1 -1.5 \times IQR$ and $Q3+ 1.5 \times IQR$, the median is depicted by a horizontal line. Pairwise comparisons of the distributions show significance assessed by a two-sided Wilcoxon rank sum test with Bonferroni multiple-test correction ($p<\alpha/m$, with $\alpha=0.05 \left( * \right), \alpha=0.01 \left( ** \right), \alpha=0.001(***)$, and NS = not significant, for $m=16$ tests). The Y-scale was adjusted (limited to 200) to remove the distortion effect of outliers on the violin plot.

**Supplementary Figure 2. Deletions between HIV-1 consensus sequences and individual HIV-1 genomes from across the world. (A)** Violin plots of the distribution of nucleotide deletions between individual HIV-1 sequences sampled up until 2002 and the 2002 consensuses (red) and individual HIV-1 sequences sampled up until 2021 and the 2021 consensuses (blue). **(B)** Violin plots of the distribution of nucleotide deletions between individual HIV-1 sequences sampled up until 2002 and the 2021 consensuses (yellow) and, again, individual HIV-1 sequences sampled up until 2021 and the 2021 consensuses (blue). Violin plot margins show the distribution of possible values, box margins 25% ($Q1$) and 75% ($Q3$) quantiles ($IQR$), box whiskers indicate $Q1 -1.5 \times IQR$ and $Q3+ 1.5 \times IQR$, the median is depicted by a horizontal line. Pairwise comparisons of the distributions show significance assessed by a two-sided Wilcoxon rank sum test with Bonferroni multiple-test correction ($p<\alpha/m$, with $\alpha=0.05 \left( * \right), \alpha=0.01 \left( ** \right), \alpha=0.001(***)$, and NS = not significant, for $m=16$ tests). The Y-scale was adjusted (limited to 150) to remove the distortion effect of outliers on the violin plot.
